# Intertwined-pulse modulation for compressive data telemetry

**DOI:** 10.1038/s41598-022-16278-0

**Published:** 2022-07-13

**Authors:** Sirous Farsiani, Amir M. Sodagar

**Affiliations:** 1 Integrated Circuits and Systems (ICAS) Lab, K. N. Toosi University of Technology, Tehran, Iran; 2 Integrated Electronics (INTELECT) Lab, EECS Department, York University, Toronto, ON Canada

**Keywords:** Electrical and electronic engineering, Biomedical engineering

## Abstract

This paper presents a novel approach for anisochronous pulse-based modulation. In the proposed approach, referred to as the *intertwined-pulse modulation* (*IPM*), every pair of consecutive symbols overlap in time. This allows for shortening the time allocated for the transmission of the symbols, hence achieving temporal compaction while the data goes through the line encoding step in a digital communication system. The IPM is also uniquely superior to other existing anisochronous pulse-based modulation schemes in the fact that it exhibits robust symbol error rate against unwanted variations in both rise/fall times of the pulses in the modulated waveform, and in the threshold level used for data detection on the receiver side. An experimental setup was developed to implement an IPM encoder using standard digital hardware, and an IPM decoder as a part of the receiver system in software. According to the experimental results (supported by simulation results and theoretical studies), for the data mean value of mid-full-scale range, the proposed IPM scheme exhibits a time-domain compaction rate of up to 209.2%.

In the era of microelectronics, exchange of data within and between a majority of electronic devices, wirelessly or through hardwired connection, is an undeniable necessity. Given the advantages digital techniques provide over their analog counterparts, digital communications is nowadays the predominant way of data transfer. As examples, one can name ultra-high-speed communications through optical fibers^1^, high data-rate chip-to-chip communications^2^, and modern generations of mobile communications dedicated to cellular phone networks^3^. From among the wide spectrum of digital modulation techniques, pulse-based schemes are known for the high data transfer rate they provide as well as the low energy they require when it comes to physical realization at the circuit (and even system) level^4,5^. Pulse-based modulation schemes are categorized, in general, into *ultra-wideband* (*UWB*) approaches and *pulse-time modulation* techniques. The former is a class of highly energy-efficient approaches, in which data is telemetered by sending pulses with extremely short widths and optimized wave shapes^6^. The latter modulates data on the timing attributes of pulses with binary amplitude levels. According to the common terminology in digital communications, pulse-time modulation techniques are categorized under *line encoding* techniques (sometimes referred to as *baseband modulation* schemes as well). In pulse-time modulation techniques, we benefit from simple hardware implementation as well as some of the intrinsic advantages of digital modulation schemes such as noise immunity^7,8^.

Based on how timing attributes of binary pulses in the modulated signal convey the data being transmitted, pulse-time modulation techniques are divided into *isochronous* and *anisochronous* categories^9^. In isochronous techniques [e.g., pulse-width modulation (PWM) and pulse-position modulation (PPM)], regardless of their contents, symbols are all allocated with the same symbol time. On the contrary in anisochronous techniques [such as pulse-interval modulation (PIM) and pulse-interval-and-width modulation (PIWM)], symbol time varies according to the content of the symbol. In the PIM scheme, the time interval between every two consecutive pulses (of the same pulse width) conveys the data being transmitted. This is while in PIWM, pulse widths are also used to carry some additional data. To conclude, a major advantage of anisochronous modulation schemes over their isochronous counterparts is the possibility of achieving enhanced transmission symbol rates.

To be able to more efficiently utilize the limited data transfer capacity of the communication channel, compression of data prior to transmission is a system-level solution. Advanced high-density neural recording brain implants are examples of such systems, acquiring huge amount of neuronal data to be telemetered to the outside world through wireless interfacing. A wide variety of digital signal processing techniques have been proposed for data compression/reduction on such devices, among which one can point to spike detection^10,11^, neural signal compression^12,13^, and spike sorting^14,15^.

In this article, a novel anisochronous pulse-time modulation scheme is introduced, which allows for time-domain compaction of the data being transmitted. The proposed scheme is a general modulation technique with a wide spectrum of applications such as optical communications, wireless digital communications, and *near-field* data telemetry to/from biomedical implants. Throughout this article, discussions and explanations are generally presented for common types of data (e.g., sine waves and random data), and as a special example for intra-cortical neural signals massively recorded and wirelessly telemetered by next-generation high-density brain implants (as a cutting-edge research forefront where compression of the data being transmitted is of utmost importance). It is worth noting that according to the common terminology in digital communications^16^, it is assumed in this article that the digital data being transmitted is a serial stream of N-bit binary words. As a result of pulse modulation, each digital word is represented by a symbol in the wave shape of the pulse modulated signal. In anisochronous pulse-time modulation schemes, length of symbols is determined by the content of the corresponding digital words.

## Results

### Intertwined-pulse modulation

In conventional pulse-time modulation techniques (both isochronous and anisochronous), symbols are encoded consecutively, meaning that encoding of the next symbol does not start before the encoding of the current symbol is complete. In this article, an anisochronous pulse-time modulation scheme is proposed, which is referred to as *Intertwined-Pulse Modulation* (*IPM*), hereafter. In this scheme, the time attributes used to encode a pair of consecutive symbols are ‘intertwined’ in such a way that the two symbols appear in the modulated signal with extensive overlap. This provides the opportunity of data transmission in a significantly more compact way compared with the other anisochronous schemes existing in the literature. In other words, in addition to performing line encoding, the proposed IPM scheme introduces the new concept of ‘*compressive’ anisochronous data telemetry* in digital communications.

**Figure 1:**
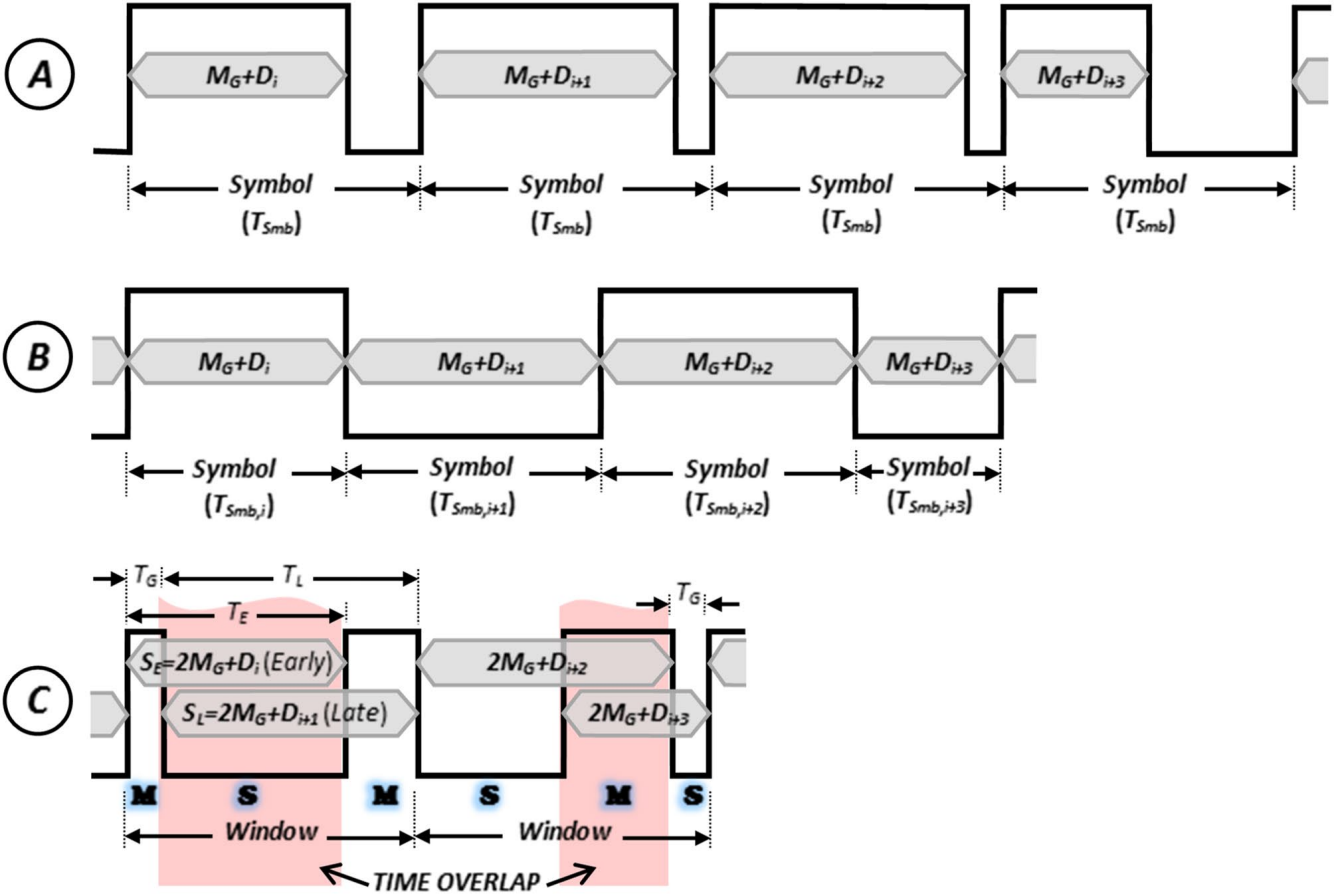
Illustration of the proposed compressive telemetry scheme in comparison with other pulse-time modulation schemes. (**a**) Pulse-width modulation, (**b**) Pulse Interval and width modulation, (**c**) Intertwined-pulse modulation (the proposed scheme).

Figure 1 illustrates how the proposed IPM scheme is realized for four consecutive data words (*D*_*i*_ ~ *D*_*i*+*3*_), and compares it with PWM (isochronous) and PIWM (anisochronous) techniques. In PWM (Fig. 1a), symbols are conveyed by a *mark*-*space* complex of fixed total length (*T*_*Smb*_), with the width of the mark modulated by the symbol data value (*D*_*i*_) plus a *guard-time coefficient* (*M*_*G*_) guaranteeing a minimum pulse width [In pulse-time modulation, ‘high’ and ‘low’ rectangular pulses conveying binary digital data are referred to as ‘mark’ and ‘space’, respectively]. The minimum width for a mark (corresponding to *D*_*i*_ = *0*) is named the *guard time*: *T*_*G*_ = *M*_*G*_*.T*_*S*_, in which *T*_*S*_ is the *slot time*, performing as the time resolution in the generation of a PWM-modulated waveform and the time associated with one least significant bit of the symbol data. The guard time, *T*_*G*_, is a constant value determined by practical constraints such as the bandwidths of the transmitter and receiver, as well as inter-symbol interference of the communication channel. In the PIWM scheme (Fig. 1b), widths of marks and spaces are both modulated by symbol data (each plus a guard-time coefficient). In the proposed IPM scheme (Fig. 1c), a complex of three alternating pulses is designated as a ‘time window’. Depending on how the previous window has ended, the time window under study is either a *mark-space-mark* (*MSM*) sequence or a *space-mark-space* (*SMS*). Each time window conveys a pair of *Early and Late symbols* (*S*_*E*_: the Early symbol, and *S*_*L*_: the Late symbol), which correspond to two consecutive digital data words, *D*_*E*_ = *D*_*k*_ and *D*_*L*_ = *D*_*k*+*1*_ (*k* = *0,2,…*). In a window of the IPM-encoded waveform, spacing of the odd edges (i.e., first and third edges) is taken as: *T*_*E*_ = *S*_*E*_.*T*_*s*_ = (*2M*_*G*_ + *D*_*E*_).*T*_*s*_, which consists of one mark and one space. Similarly, the time interval between the even edges (i.e., second and fourth edges) in the encoded waveform is equal to *T*_*L*_ = (*2M*_*G*_ + *D*_*L*_).*T*_*s*_.

Regardless of whether the time window is MSM or SMS, the IPM modulation procedure (i.e., the procedure for the generation of the IPM waveform) can be explained as follows: A time window always starts with the start of the early symbol (i.e., the 1st edge), and ends with the end of the late symbol (i.e., the 4th edge). As illustrated in Fig. 1c, length of the early symbol (*T*_*E*_) determines where the 3rd edge occurs. Timing of the 2nd edge, however, depends on which one of the early and late symbols are longer. If the late symbol is longer than the early one, it will start by a minimum clearance of *T*_*G*_ from the first edge, and will end by *T*_*L*_ after the 2nd edge. In other words, the 2nd edge in this case occurs by only one guard time after the 1st edge. Otherwise, if the early symbol is longer than the late one, the late symbol will end by *T*_*G*_ after the end of the early symbol. Length of the late symbol therefore determines where the start of it (i.e., the 2nd edge) would be. Data demodulation on the receiver side is as simple as measuring the time interval between the odd and even edges in the detected binary waveform.

### Overlapping symbols

**Figure 2:**
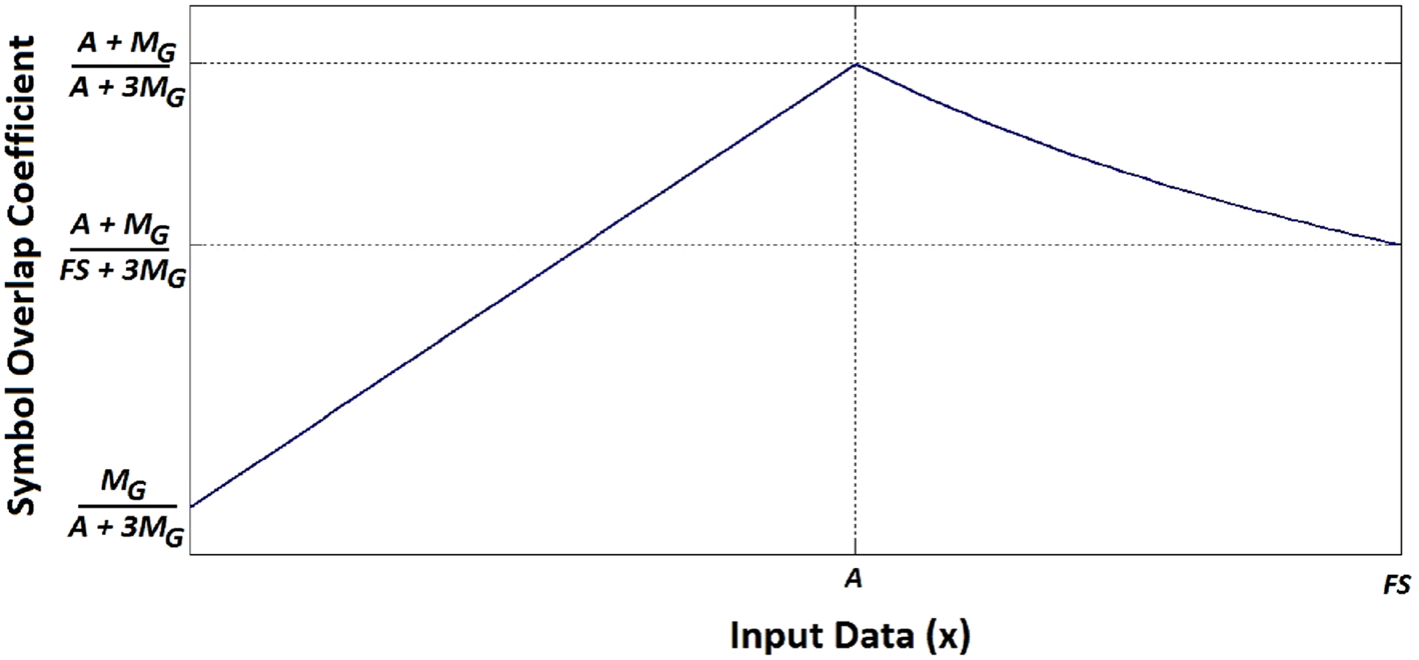
The symbol overlap coefficient, OC_Smb_, when one of the data words equals A and the other one spans the range of 0 to FS.

It was mentioned that superiority of the proposed scheme over the existing anisochronous pulse-base modulation schemes stems in the time overlap of the early and late symbols in a time window. As a measure for the extent of time overlapping in the proposed scheme, the *symbol overlap coefficient* (*OC*_*Smb*_) is hereby defined as:

1 $$ OC_{Smb} = \frac{{T_{OL} }}{{T_{Window} }} = \frac{{\min \left\{ {D_{E} ,D_{L} } \right\} + M_{G} }}{{max\left\{ {D_{E} ,D_{L} } \right\} + 3M_{G} }} $$

in which $T_{Window}$ denotes the window width, and $T_{OL}$ is the extent of the time overlap between the early and late symbols in the window. Figure 2 plots OC_*Smb*_ when the data word associated with one of the symbols in an IPM time window equals an arbitrary value of *A*, and that of the other symbol spans the entire data range (i.e., 0 to the full-scale value, *FS*). According to this graph, for a pair of early and late symbols in an IPM window, OC_*Smb*_ is maximized when the two symbols are equal in amplitude (i.e., *D*_*E*_ = *D*_*L*_ = *A*). The absolute maximum OC_*Smb*_ is, therefore, achieved when the amplitudes of the two symbols are both at the full-scale level (*FS*):

2 $$ OC_{Smb} \left( {max} \right) = \frac{{FS + M_{G} }}{{FS + 3M_{G} }} $$

**Figure 3:**
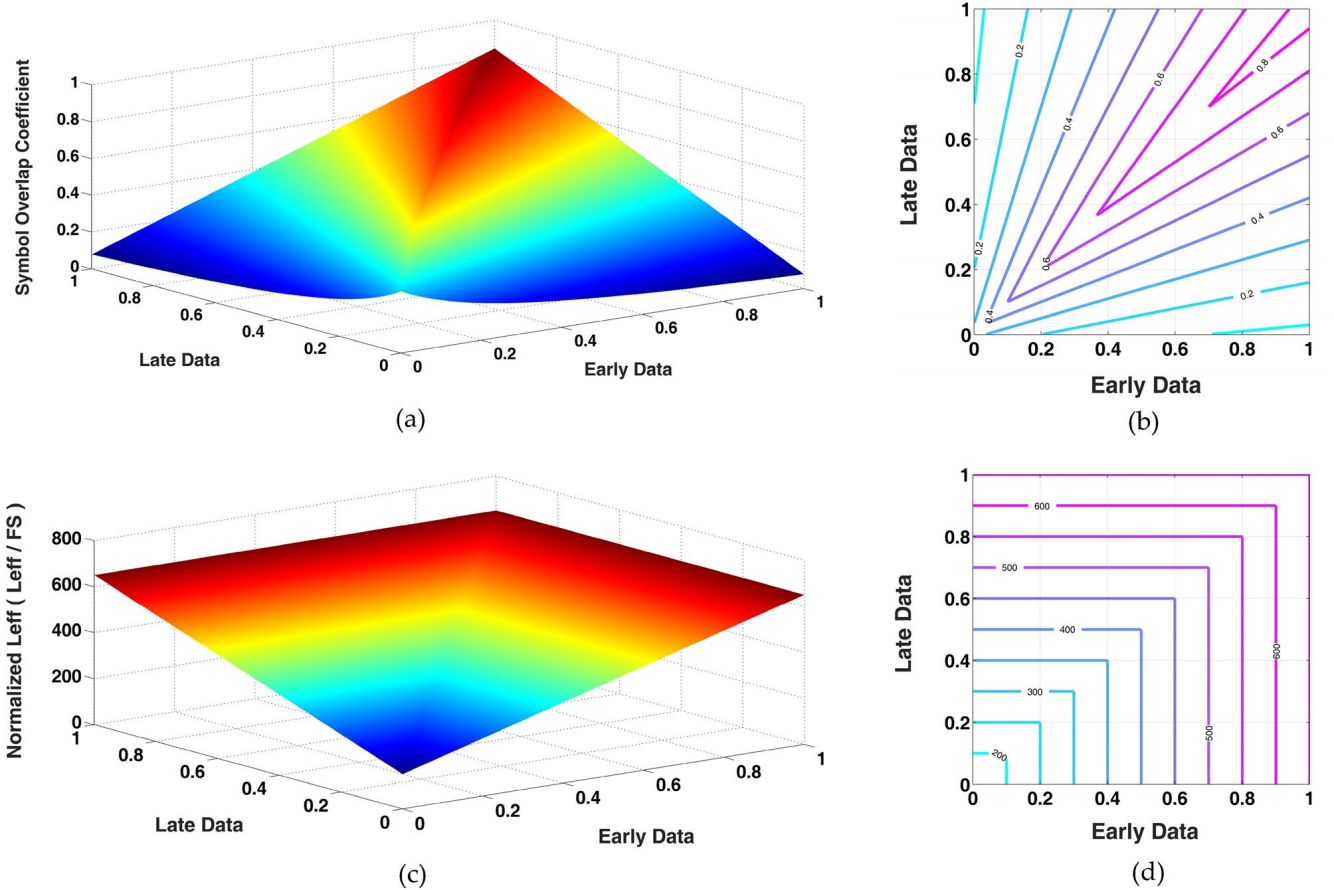
Contribution of the length of early and late symbols to symbol overlap and symbol length in IPM coding. (**a**) Symbol overlap coefficient for all possible combinations of two data samples, (**b**) the contour graph (values on the graph represents the corresponding OC_*Smb*_ on each line) for the 3D plot in part a, (**c**) normalized effective symbol length of IPM-coded signal for all possible combinations of two data samples, and (**d**) the contour graph (values on the graph represents the corresponding *L*_*eff*_ on each line) for the 3D plot in part.

The largest achievable value for OC_*Smb*_(max) is as high as ~ 100%, corresponding to the design case where the guard time coefficient is much smaller than the full-scale symbol amplitude (i.e., *M*_*G*_ <  < *FS*). The overlap coefficient for all possible combinations of two consecutive signal samples is shown in Fig. 3a,b in the form of both a 3-dimentional surface and a contour graph. Equation (1) defines the instantaneous overlap coefficient for pair of early and late symbols in a time window. The overall overlap coefficient over the entire course of the signal is introduced as:

3 $$ OC = \frac{{\sum {\text{T}}_{{{\text{OL}},{\text{i}}}} }}{{\sum {\text{T}}_{{{\text{Window}},{\text{i}}}} }} $$

### Average symbol length

Since each time window in the proposed IPM scheme is used to convey two symbols, we define the *effective symbol length*, *L*_*eff*_, as half the window length:

4 $$ L_{eff} = \frac{{T_{Window} }}{2} = \frac{1}{2}\left( { 3M_{G} + Max\left\{ {D_{E} ,D_{L} } \right\}} \right).T_{s} $$

Figure 3c,d plots the effective symbol length for all possible early and late data values, in which the data values as well as the effective symbol length are all normalized to the full-scale value (FS), and the guard time coefficient is taken equal to FS/10. This plot indicates that the effective symbol length becomes significantly shorter when the early and late data words are both on the lower side of the amplitude range. As derived in *Methods*, assuming that the data words take on random values within the range of 0 to FS with uniform distribution (usually used as a reference benchmark in pulse-based modulation^17^), the *average symbol length* for an IPM-encoded signal is derived as:

5 $$ L_{avg,IMP} = E\left[ {L_{eff} } \right] = \frac{1}{2}\left( {3M_{G} + \frac{2}{3}FS} \right).T_{s} $$

in which *E*[.] is the expected value operator.

### Data compaction in the time domain

**Figure 4:**
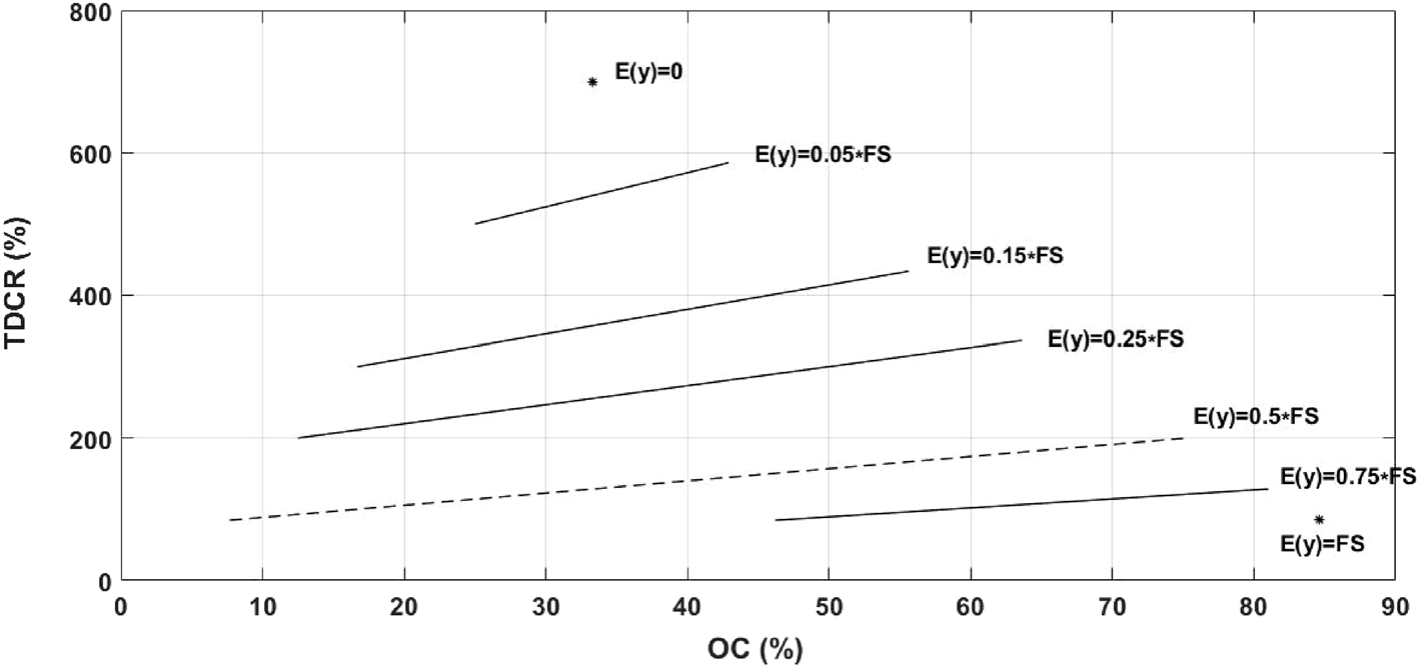
TDCR-OC loci for signals with different expected values (for M_G_ = 0.1FS).

The variable-time coding that is intrinsic to all anisochronous pulse modulation techniques as well as the time overlap that exists in the definition of the proposed IPM scheme both contribute to achieving time-domain compaction of the data being telemetered. We hereby introduce *time-domain compression rate* (*TDCR*) to quantify the effective performance of anisochronous PTM techniques in symbol-length reduction referenced to the isochronous PWM technique (with no compaction) for the same data:

6 $$ TDCR\% = \left( {\frac{{L_{avg,PWM} }}{{L_{avg} }} - 1} \right) \times 100\% $$

in which $L_{avg}$ is the average symbol length for the modulation technique under study and $L_{avg,PWM}$ is that for a PWM-coded signal. As derived in *Methods*, aside from the contribution of the design parameters *FS* and *M*_*G*_, TDCR is a linear function of the overall overlap coefficient:

7 $$ TDCR_{IPM} \% = \left[ {\frac{{\left( {2M_{G} + FS} \right).OC}}{{2M_{G} + E\left( {Data} \right)}} + \frac{{FS - E\left( {Data} \right)}}{{2M_{G} + E\left( {Data} \right)}}} \right] \times 100\% $$

in which *E*(*Data*)) is the expected value of the data. Figure 4 plots the TDCR-OC loci for the IPM scheme for different data expected values. It is worth noting that for a given signal, only one pair of (OC, TDCR) values is achieved, which is designated as one single point on the TDCR-OC plot. Smaller signal expected values help shorten the average symbol length, hence achieving higher TDCR. Furthermore, for a given signal expected value, the more the extent of the symbol overlap is, the higher added time-domain compaction is achieved.

**Figure 5:**
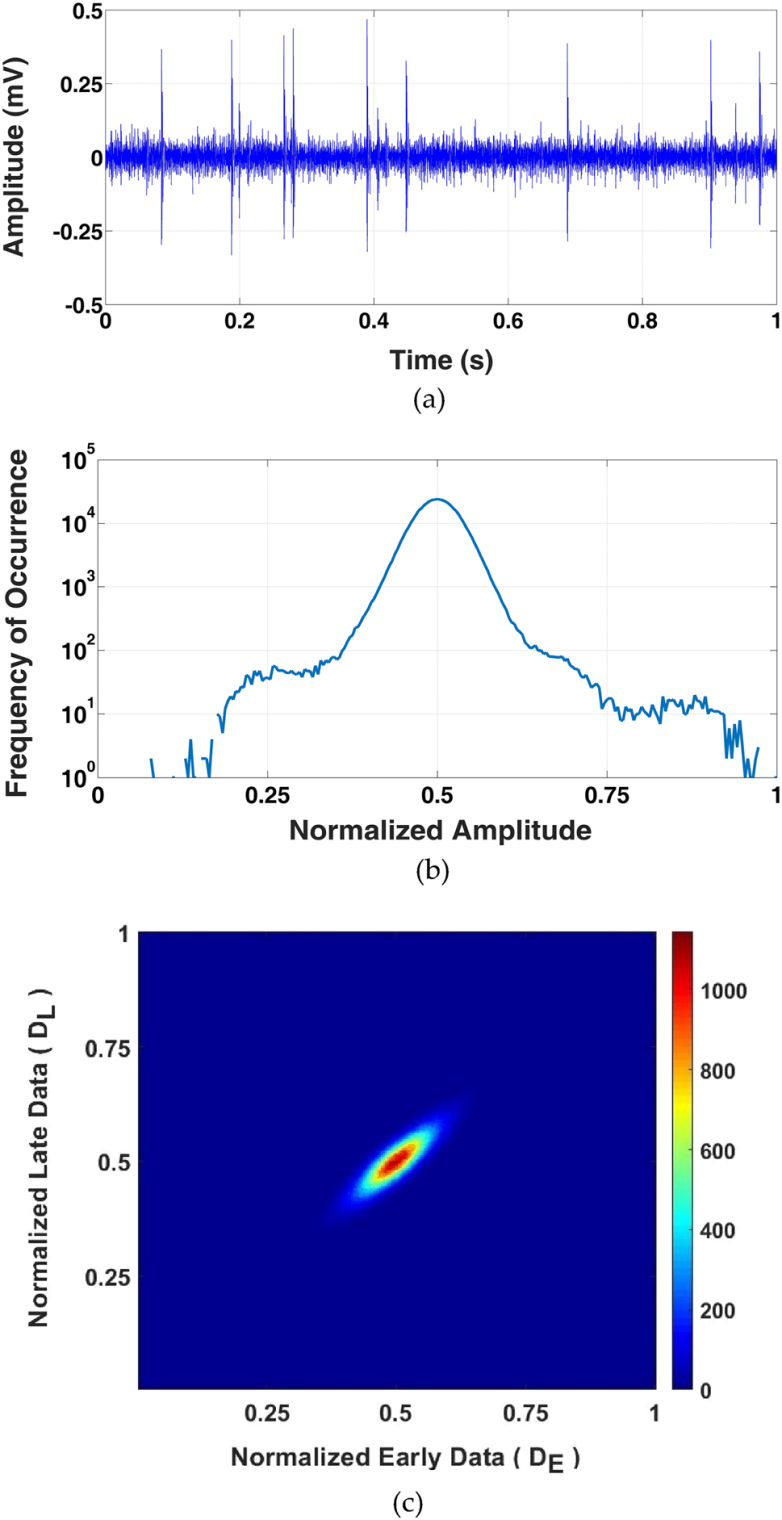
An intra-cortically recorded neural signal as an example of the type of signals, for which the IPM scheme exhibits high compaction efficiency. (**a**) The signal in the time domain, (**b**) signal histogram, and (**c**) density plot of binary combination of this data.

According to Fig. 3a,b and Eq. (7), higher OC and consequently greater TDCR is achieved when the early and late signal samples in an IPM time window are close in value. Therefore, for deterministic signals sampled at sufficiently higher than the Nyquist rate (> 10*f*_*Max*_), variations of the signal amplitude are rather smooth and satisfy the implied precondition for achieving a high OC. Even for random signals, provided that the probability density function is concentrated within a rather small amplitude range (e.g., the Gaussian distribution with small standard deviation) significantly large OC and consequently high time-domain compaction is achieved. With the same reasoning, the IPM scheme also results in high time-domain compaction for the type of signals whose amplitude usually take on values around a certain baseline level, either of constant value or slowly varying with time. Biological signals such as electrocardiograms and intracortically-recorded neural signals can be named as examples of such signals. As a sample signal exhibiting this property, Fig. 5a presents a 1-s. intra-cortical, extra-cellular neural signal in the time domain, recorded *in-vivo* from the auditory cortex of a guinea pig with a resolution of 8 bits sampled at a rate of 20 k.samples/s^18^. For this recording, which comprises action potentials and background noise with a signal-to-noise ratio of 14.1 dB. Parts *b* and *c* of Fig. 5 show the associated (logarithmically-scaled) amplitude distribution and data density plot for consecutive sample pairs (to be used as early and late samples in an IPM waveform), respectively. The distribution peak in Fig. 5b mainly corresponds to the background noise fluctuations around the signal baseline (at FS/2). Being within a rather small amplitude range, every two consecutive samples of the background noise, therefore, are most likely located on (or around) the bisector of the plot of Fig. 3b, and consequently achieving a relatively large OC value. The less frequent signal amplitudes on the two sides of the distribution represent the large amplitude variations associated with neural spikes (also known as action potentials). Every two consecutive samples on the rather smoothly-varying neural spikes are also close enough in amplitude to be projected on (or around) the bisector of the plot in Fig. 3b, and again result in a rather large OC value. In the data density plot of Fig. 5c, concentration of the early-late sample pairs along the bisector demonstrates the potential of achieving rather high OC for this type of signal, if modulated using the IPM scheme (as will be reported in *Experimental Results*).

### Symbol error rate

As it is common in digital communications, efficacy of the proposed modulation scheme in terms of the transported data integrity is evaluated by presenting the rate of symbol errors. Commonly used as a performance measure, *symbol error rate* is, in general, a function of the following three contributors: (1) the noise accompanying the signal upon arrival at the receiver end as well as the noise generated inside the receiver circuit, (2) strength of the received signal, and (3) deviation of the threshold level used for the detection of the received data from its optimum value.

**Figure 6:**
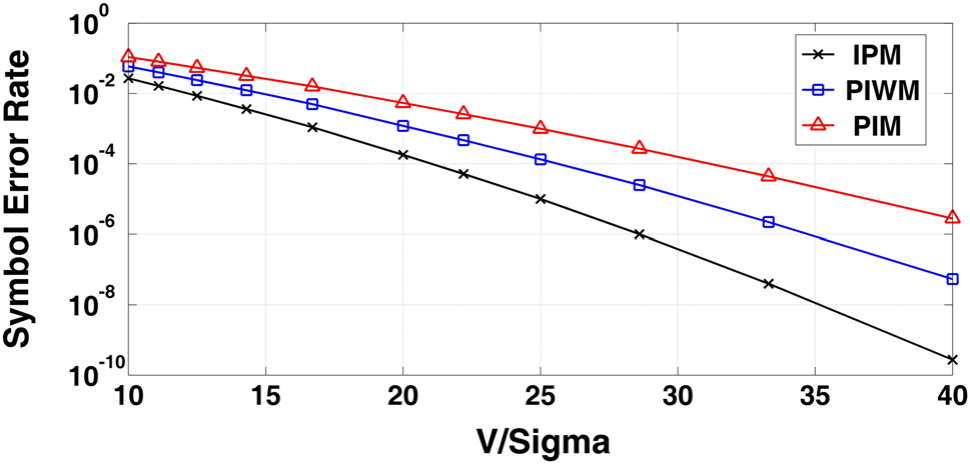
Symbol-error rate for IPM, PIWM and PIM (symbol rate = 5 MS/s , *T*_*r*_ = *T*_*f*_ = 4 ns).

Generally, in the IPM scheme, noise can appear on both amplitude and timing of the modulated waveform. As it is the case for other pulse-based modulation schemes, rather large amplitude noise can possibly lead to logic level detection error on the receiver side. However, similar to other anisochronous techniques, envision of sufficiently long guard times helps reduce the chance of this kind of error. The noise added to the pulse edge timing, also known as *jitter*, limits the effective resolution of the pulse widths in an IPM signal and consequently the maximum achievable symbol rate. It should be added that the pulse smearing caused by the limited bandwidth of the transmitter and receiver circuits is a factor that can possibly worsen the effect of the jitter. To conclude, the IPM scheme is superior in noise performance to other anisochronous modulation schemes mainly because of its compression advantage. In other words, at the same symbol rate, the pulse times allocated to symbols in an IPM signal are longer than those for any other anisochronous scheme. From the perspective of noise performance, this is translated to relatively less susceptibility to timing jitter, which results in lower probability of symbol error for the IPM approach. Figure 6 presents the symbol error rate for the IPM scheme as well as that for PIWM and PIM schemes as a function of *V*_*H*_*/σ* (as a measure for signal-to-noise ratio). In this plot, *V*_*H*_ is the high logic level (the low logic level is assumed to be *V*_*L*_ = 0 V), and $\sigma$ is the standard deviation of the noise, which is assumed to be of Gaussian distribution and with a white power spectrum. It should be added that in this study, the input signals are of a symbol rate of 5 M symbols/s. and with rise and fall times of $T_{r} = T_{f} = 4\;ns$.

**Figure 7:**
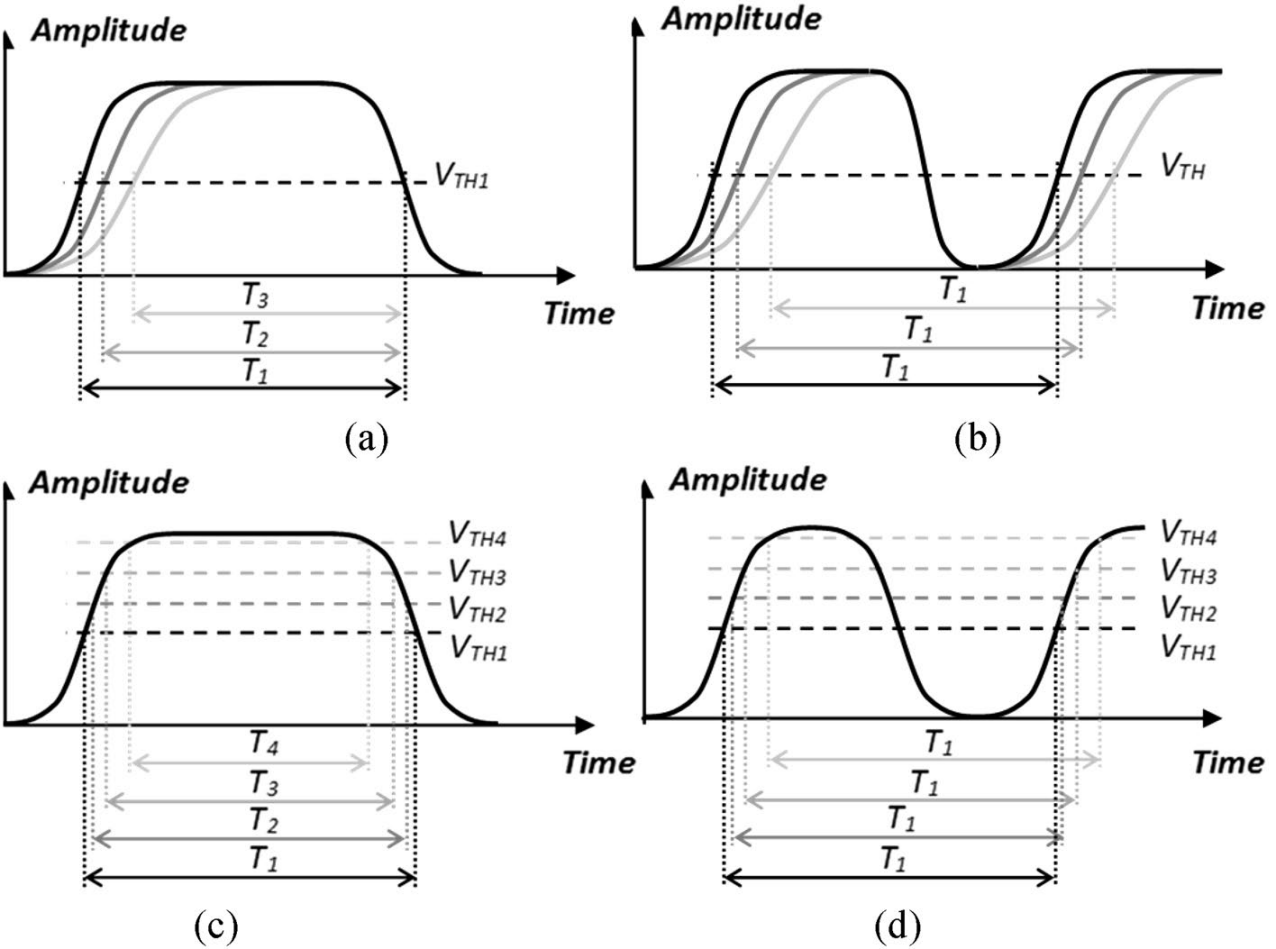
Illustration of the superiority of the IPM scheme to other anisotropic modulation schemes in terms of resilience against systematic imperfections in pulse widths and upon pulse-width measurement. (**a**) Non-equal rise/fall times cause pulse-width error in regular anisotropic modulation schemes (e.g., PIM and PIWM). (**b**) In the proposed scheme (IPM) pulse-width is not affected by the difference between rise and fall times. (**c**) Contribution of the detection threshold level to symbol error in regular anisotropic modulation schemes. (**d**) Robustness of the proposed scheme (IPM) against threshold level deviations.

In reality, functional and practical non-idealities and limitations in physical systems (e.g., transmitter and receiver circuits as well as the communication channel) cause the rising and falling edges in a binary pulse to take non-zero times (*T*_*r*_ and *T*_*f*_ ≠ 0), with no guarantee to be identical (*T*_*r*_ ≠ *T*_*f*_). As illustrated in Fig. 7a, in other anisochronous modulation schemes, the difference between rise and fall times introduces a non-zero error in the symbol time, which evidently leads to the degradation of the symbol error rate. The proposed IPM scheme, however, does not suffer from this phenomenon as the symbols are defined between two pulse edges of the same type (being either HL or LH). This is because, as shown in Fig. 7b, any non-ideality the first edge is subject to (affecting the symbol timing usually in the form of edge displacements such as non-zero transition time and propagation delay), also happens to the second edge of the same type. Therefore, the symbol time and consequently the symbol error rate remain unaffected.

Existence and also widths of the pulses arrived at the receiver end are traditionally detected based on hard-thresholding and time to digital conversion^8^. In the majority of pulse-based modulation schemes (e.g., PWM, PIM, and PIWM), symbols are modulated onto the width of pulses. In practice, the detection threshold level can deviate from its optimal value for reasons such as variations in the detected signal strength, threshold generation error, or noise. Confined between a pair of rising and falling edges with non-zero transition times, measured width of a received pulse in the aforementioned schemes is undeniably affected by any deviation in the threshold value. Consequently, as illustrated in Fig. 7c, this can cause error in the detection of the received symbol. Another unique property of the proposed IPM scheme is that the measurement of the received pulse widths is insensitive to deviations in the threshold level. This is due to the fact that (as shown in Fig. 7d) in an IPM signal, symbols are confined between two consecutive edges of the same type, either rising or falling.

## Experimental results

**Figure 8:**
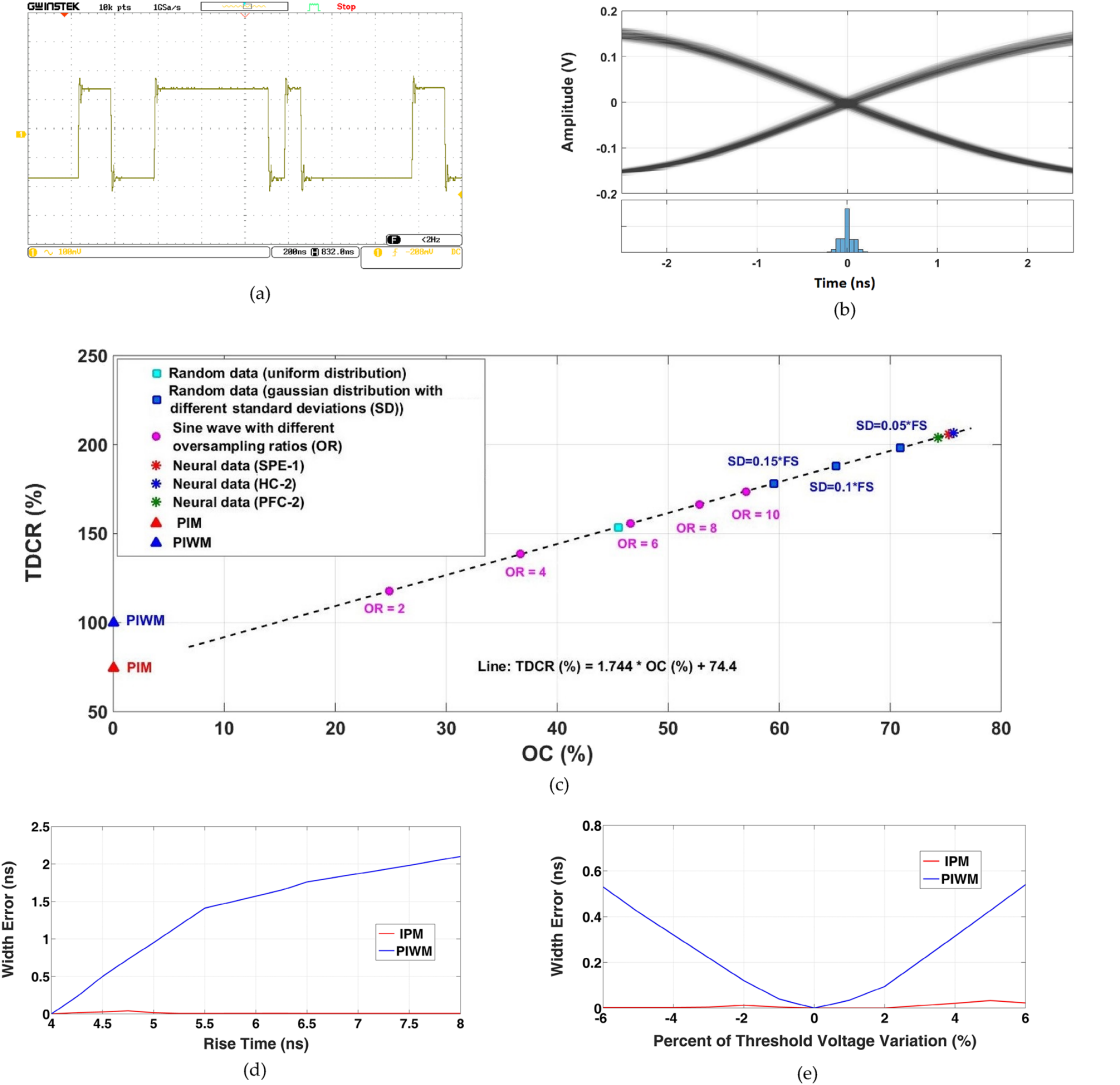
Experimental verification and characterization of the key properties of the IPM scheme and comparison with other anisochronous schemes. (**a**) oscilloscope screen shot showing an IPM-encoded waveform, (**b**) aligned rising and falling edges for 3000 IPM pulses on the receiver side of the experimental setup as well as the associated pulse-width error histogram, (**c**) the TDCR-OC plot for the IPM scheme in the case of sinewaves (with oversampling ratios ranging from 2 to 10), random data with uniform and Gaussian distributions (for the Gaussian case, standard deviations of 5%, 10%, and 15% of the full-scale amplitude), and three different intra-cortical neural signals. It should be noted that all the same signals were encoded on the same setup with PIM and PIWM approaches, the results of which are exactly on the same spots designated using triangular symbols, (**d**) effect of rise time variation on the width error for IPM and PIWM pulses (fall time is fixed and equal to 4 ns), and (**e**) effect of threshold voltage variation on width error for IPM and PIWM pulses.

In order to verify and evaluate the performance of the proposed technique, an experimental setup realizing a hardwired digital communication system is prototyped. Implemented in hardware on a Xilinx S6LX9 FPGA, transmitter side of the setup includes an IPM encoder, as well as three other encoders for PIWM, PIM, and PWM schemes. Following the encoding step, there is a line driver, and the bandwidth of the line (communication channel) can be programmed within the range of 10–70 MHz. Operating at a clock rate of up to 320 MHz, the encoders can be operated with configurable slot time. Receiver side of the setup first captures the line voltage at a sample rate of 1 GS/s, and then implements a data detector, an IPM decoder, as well as decoders for PIWM, PIM, and PWM schemes in software. The data detector works based on hard-thresholding, with a tunable threshold level. To enhance the detection resolution in the time domain, hard-thresholding of the signal level is performed on an interpolated version of the received samples. With measured jitter of around 57 ps for the IPM pulse timings and rise and fall times of 4 ns (for the bandwidth of 70 MHz), slot time of the IPM encoder (as well as that for other encoders) is set at 3.125 ns. At a clock rate of 320 MHz, the guard time in this experiment is set to 68.75 ns (equivalent to a guard time coefficient *M*_*G*_ of 22) in order to avoid inter-symbol interference. A sample IPM waveform captured at the receiver end of the communication channel with a bandwidth of 70 MHz is shown in Fig. 8a. Variability of the timings of the rising and falling edges for 3000 captured pulses and the associated error histogram are presented in Fig. 8b.

The experimental setup in this work is used to IPM-encode 100 channels of intra-cortical neural signals recorded from primary motor and somatosensory cortex of rats using a 384-channel Neuropixel probe^19^. Named as the ‘SPE-1’ dataset, the signals have a resolution of 10 bits (reduced to 8 bits for this work), and are sampled at a rate of 30 k samples per second on each channel. Operating at a rate of 3 M.Symbols/sec., and with a measured symbol error rate of better than 10^–11^, the achieved overlap coefficient and time-domain compaction rate are 75.7% and 206.5%, respectively.

Demonstrating the impact of the data on the time-domain compaction achieved through the IPM scheme, the prototyped IPM encoder was also fed with sinewaves (with different oversampling ratios), and random signals with uniform and Gaussian distributions (with different standard deviations). Figure 8c compares the experimental results achieved for all the input signals on the same TDCR-OC plot. Having the same mean value of FS/2, TDCR and OC values for all the signals are projected on a straight line as modeled by Eq. (7) and plotted in Fig. 4. As theorized before, as the oversampling ratio for a sine wave increases, higher OC and consequently greater TDCR values are achieved. So long as the data values are confined within the 0 to full-scale range (with no truncation), a random signal with Gaussian distribution results in better TDCR-OC performance compared with a uniformly-distributed random signal, and that is because of the concentration it has around the mean value. As expected, for Gaussian random signals, the smaller the standard deviation is, the compression performance point will climb higher on the TDCR-OC plot. Compared with all other types of input signals, the neural signal exhibits the best compaction performance as it is highly concentrated around the FS/2 baseline. It is worth noting that two other intra-cortical neural signals (recorded from the prefrontal cortex (PFC-2)^20^ and hippocampus of rats (HC-2)^21^) normalized to the same statistical specifications (i.e., full-scale amplitude range and mean value) demonstrate almost the same TDCR-OC results (with a maximum deviation of ± 3%). The deviation in the results in this case is believed to be because of the different signal-to-noise ratios and firing rates of the signals.

In the experimental setup, PIM and PIWM encoders are also implemented using standard digital hardware. Those encoders are tested using the same input signals for fair comparison, the results of which are also shown on the TDCR-OC plot of Fig. 8c. As expected, regardless of the type or pattern of the data, the OC is always zero for PIM and PIWM techniques. Moreover, given the fact that the time-domain compaction achieved using those techniques is merely a function of the signal mean value, the TDCR achieved by those techniques does not vary by changing the input signal. To conclude, for a mean value of *FS*/2, the IPM scheme is capable of enhancing the time-domain compaction rate to up to 209.2%. According to Eqs. (1) and (7), this enhancement easily grows up to 296.4%, 361.3%, and 411.7% if the signal mean value is lowered to FS/3, FS/4, and FS/5, respectively.

Figure 8d compares the average width error for the IPM scheme with that for PIWM. In this test, the rise time is swept from 4 to 8 ns while the fall time is kept constant at *T*_*f*_ = 4 ns. While for PIWM the width error increases with the difference between the rise and fall times, the IPM pulse width is not affected by changes in pulse transition times (i.e., *T*_*r*_ or *T*_*f*_). According to the results plotted in Fig. 8d, while the width error is kept well below 50 ps for IPM, this error for PIWM increases up to more than 2 ns.

Figure 8e proves the advantage of the IPM scheme over PIWM when the threshold level is subject to ± 6% deviation with respect to the optimal thresholding value. While the average width error for PIWM grows to more than 0.5 ns, that for the IPM scheme does not exceed 40 ps. The same results are achieved when the threshold voltage is kept constant and the amplitude of the received signal varies.

## Discussion

In this article, the *intertwined pulse modulation* (*IPM*) scheme is proposed, which is of significantly higher time-domain compaction performance compared to other anisochronous pulse modulation techniques. The proposed scheme adds the time overlapping property to the ‘variable symbol length’ nature of anisochronous techniques. This introduces IPM as a *compressive data telemetry* approach, which performs temporal data compression while the data passes through the traditional chain of steps in a regular digital communication system; hardwired or wireless. This is totally independent of and in addition to any other temporal or spatial compression technique that might have been applied on the data in the source coding step (i.e., prior to data transmission). Supported by both theoretical derivations and experimental results, the IPM scheme is capable of exhibiting significantly higher time-domain compaction rates compared with the best anisochronous modulation technique in the literature (*i.e.*, PIWM) and at the same time remarkably lower symbol error performance.

To design the IPM scheme for high-rate data transmission, one needs to choose small-enough slot time and guard time. The minimum possble slot time, in a practical scenario, is determined by the maximum speed of the IPM modulator and demodulator circuits. Rapid prototyping solutions such as FPGAs can be used for low to medium bit rates (i.e., up to around 40 Mbps using state-of-the-art devices). For high bit rates (i.e., around 400 Mbps and higher), transmitter and receiver circuitry need to be implenmented on custom-designed application-specific integrated circuit (ASIC) chips. The choice of the guard time is usually determined by both the noise quality of the channel and the performance of the receiver circuitry (especially the data detector block). When designing for extremely high data rates, we should be aware of the tradeoff between the data rate and the symbol error rate (as it is the case for other similar schemes as well).

Main aim of the proposed IPM scheme is providing the possibility of data compaction. This is not necessarily translated into achieving extremely higher bit rates. What it means is that we are now significantly more “bit rate efficient”. Given the trade off that exists between the bit rate on one hand and the bit error rate (BER) and power consumption on the other hand, this can be interpreted in two ways: (a) Spending the same electric power and with the same BER, the IPM scheme can significantly elevate the bit rate, and (b) Staying at the same bit rate, we can benefit from more relaxed time constraints in data transmission. As a result, at the same data rate, the IPM scheme offers significant improvement (reduction) in BER as well as considerable saving in dynamic power consumption.

Finally, the IPM scheme can be combined with other existing techniques in order to enhance the performance of data transmission. The combination of IPM and ultra-wideband (UWB) techniques can be named as an example.

## Methods

### Time-domain representation of an IPM signal

To derive a general representation for an IPM signal for the *i*-th window in the time domain, let us assume that the two consecutive data words, *D*_*2i*_ and *D*_*2i*+*1*_, are encoded using a sequence of three pulses, *P*_*3i*_, *P*_*3i*+*1*_, and *P*_*3i*+*2*_, being either Mark-Space-Mark or Space-Mark-Space. The procedure introduced in *Results (*to correspond the early and late data values to the timing attributes of the associated time window) can be expressed in mathematical terms as:

8a $$ S_{E} = \left( {P_{3i} + P_{3i + 1} } \right) = \left( {2M_{G} + D_{2i} } \right).T_{S} , $$

8b $$ S_{L} = \left( {P_{3i + 1} + P_{3i + 2} } \right) = \left( {2M_{G} + D_{2i + 1} } \right).T_{S} , $$

and

8c $$ (P_{3i} + P_{3i + 2} ) = \left( {2M_{G} + \left| {D_{2i} - D_{2i + 1} } \right|} \right).T_{S} $$

According to Eqs. (8a–8c), the widths of the three pulses can be written in terms of both the values of the associated data words and the guard time coefficient, *M*_*G*_, as:

9a $$ P_{3i} = \left\{ {\begin{array}{*{20}c} {M_{G} .T_{S} } & {D_{2i} < D_{2i + 1} } \\ {\left( {M_{G} + D_{2i} - D_{2i + 1} } \right).T_{S} } & {D_{2i} \ge D_{2i + 1} } \\ \end{array} } \right., $$

9b $$ P_{3i + 1} = \left\{ {\begin{array}{*{20}c} {\left( {M_{G} + D_{2i} } \right).T_{S} } & {D_{2i} < D_{2i + 1} } \\ {\left( {M_{G} + D_{2i + 1} } \right).T_{S} } & {D_{2i} \ge D_{2i + 1} } \\ \end{array} } \right., $$

and

9c $$ P_{3i + 2} = \left\{ {\begin{array}{*{20}c} {\left( {M_{G} + D_{2i + 1} - D_{2i} } \right).T_{S} } & {D_{2i} < D_{2i + 1} } \\ {M_{G} .T_{S} } & {D_{2i} \ge D_{2i + 1} } \\ \end{array} } \right.. $$

The general form for an IPM data window conveying two symbols can be expressed as:

10a $$ W_{i} \left( t \right) = \left\{ {\begin{array}{*{20}c} {\prod \left( {\frac{{\frac{t}{{T_{s} }} - \left( {P_{3i} + \frac{1}{2}P_{3i + 1} } \right)}}{{P_{3i + 1} }}} \right)} & {:SMS\;window} \\ {\prod \left( {\frac{{\frac{t}{{T_{s} }} - \frac{1}{2}P_{3i} }}{{P_{3i} }}} \right) + \prod \left( {\frac{{\frac{t}{{T_{s} }} - \left( {P_{3i + 1} + P_{3i} + \frac{1}{2}P_{3i + 2} } \right)}}{{P_{3i + 2} }}} \right)} & {:MSM\;window} \\ \end{array} } \right. $$

in which $\prod \left( . \right)$ is the rectangular pulse function, and $T_{s}$ is the slot time. The IPM signal in the time domain is, therefore, formulated as:

10b $$ x_{IPM} \left( t \right) = \mathop \sum \limits_{i = - \infty }^{\infty } W_{i} \left[ {t - T_{s} \mathop \sum \limits_{a = - \infty }^{3i - 1} \left( {p_{i} } \right)} \right] $$

### The average IPM symbol length

Based upon the time-domain representation derived for an IPM signal in Eq. (10), the average symbol length for uniformly-distributed random data (as a common benchmark) over the range of 0 to the full-scale value of *FS* is achieved by calculating the expected value of the effective symbol length as:

$$ \begin{aligned} L_{avg,IPM} \left( i \right) & = E\left[ {L_{eff} } \right] \\ & = \frac{1}{2}E\left[ {\left( {3M_{G} + Max\left\{ {D_{2i} ,D_{2i + 1} } \right\}} \right).T_{s} } \right] \\ & = \frac{1}{2}\left( {3M_{G} + E\left[ {\max \left\{ {D_{2i} ,D_{2i + 1} } \right\}} \right]} \right).T_{s} \\ & = \frac{1}{2}\left( {3M_{G} + \smallint max\left( {x,x1} \right).f_{X} \left( x \right) dx} \right).T_{s} \\ & = \frac{1}{2}\left( {3M_{G} + \frac{1}{FS}\mathop \smallint \limits_{0}^{FS} max\left( {x,x1} \right)dx} \right). \\ & = \frac{1}{2}\left( {3M_{G} + \frac{1}{FS}\mathop \smallint \limits_{0}^{FS} \left[ {P\left( {x > x1} \right).x + P\left( {x1 > x} \right).\left( {\frac{FS + x}{2}} \right)} \right] dx} \right). T_{s} \\ & = \frac{1}{2}\left( {3M_{G} + \frac{1}{FS} \mathop \smallint \limits_{0}^{FS} (\frac{x}{FS}.x + \frac{FS - x}{{FS}}.\frac{FS + x}{2}} \right) dx).T_{s} \\ & = \frac{1}{2}(3M_{G} + \frac{1}{FS} \mathop \smallint \limits_{0}^{FS} \frac{{FS^{2} + x^{2} }}{2FS} dx) .T_{s} \\ \end{aligned} $$

Finally, the average symbol length for an IPM-coded signal (in the case of uniformly-distributed random data) is achieved as:

11 $$ L_{avg,IMP} = E\left[ {L_{eff} } \right] = \left( {\frac{3}{2}M_{G} + \frac{1}{3}FS} \right).T_{s} $$

### Equivalent bandwidth

The equivalent bandwidth for pulse-based modulation techniques is determined as 1/*T*_*S*_. Finding *T*_*S*_ from (11), and replacing the average symbol length by the inverse of the average symbol rate, *R*_*S*_*,* equivalent bandwidth for the IPM scheme is achieved as:

12 $$ BW_{IPM} = \left( {\frac{3}{2}M_{G} + \frac{1}{3}FS} \right).R_{s} . $$

Expressing the average symbol rate *R*_*s*_ in terms of the bit rate, *R*_*b*_, (i.e., Rs = Rb/$\log_{2} FS$), Eq. (12) can be rewritten as:

13 $$ BW_{IPM} = \frac{{\left( {\frac{3}{2}M_{G} + \frac{1}{3}FS} \right)}}{{\log_{2} FS}}.R_{b} $$

and the *bandwidth utilization efficiency* for the IPM scheme is achieved as:

14 $$ \eta_{BU} = \frac{{R_{b} }}{{BW_{IPM} }} = \frac{{\log_{2} FS}}{{\frac{3}{2}M_{G} + \frac{1}{3}FS}} $$

## Data Availability

The data sets used for this paper are publicly available at their addresses given in the references section. The Matlab codes used for analysis is also available upon reasonable request.
